# Can We Use Antibodies to *Chlamydia trachomatis* as a Surveillance Tool for National Trachoma Control Programs? Results from a District Survey

**DOI:** 10.1371/journal.pntd.0004352

**Published:** 2016-01-15

**Authors:** Sheila K. West, Beatriz Munoz, Jerusha Weaver, Zakayo Mrango, Laura Dize, Charlotte Gaydos, Thomas C. Quinn, Diana L. Martin

**Affiliations:** 1 Dana Center for Preventive Ophthalmology, Johns Hopkins University, Baltimore, Maryland, United States of America; 2 National Institute for Medical Research, Kilosa, Tanzania; 3 Division of Infectious Diseases, Johns Hopkins University, Baltimore, Maryland, United States of America; 4 Division of Intramural Research, National Institute of Allergy and Infectious Diseases, NIH, Bethesda, Maryland, United States of America; 5 Centers for Disease Control and Prevention, Atlanta, Georgia, United States of America; University of Cambridge, UNITED KINGDOM

## Abstract

**Background:**

Trachoma is targeted for elimination by 2020. World Health Organization advises districts to undertake surveillance when follicular trachoma (TF) <5% in children 1–9 years and mass antibiotic administration has ceased. There is a question if other tools could be used for surveillance as well. We report data from a test for antibodies to *C*. *trachomatis* antigen pgp3 as a possible tool.

**Methodology:**

We randomly sampled 30 hamlets in Kilosa district, Tanzania, and randomly selected 50 children ages 1–9 per hamlet. The tarsal conjunctivae were graded for trachoma (TF), tested for *C*. *trachomatis* infection (Aptima Combo2 assay: Hologic, San Diego, CA), and a dried blood spot processed for antibodies to *C*. *trachomatis* pgp3 using a multiplex bead assay on a Luminex 100 platform.

**Principal findings:**

The prevalence of trachoma (TF) was 0.4%, well below the <5% indicator for re-starting a program. Infection was also low, 1.1%. Of the 30 hamlets, 22 had neither infection nor TF. Antibody positivity overall was low, 7.5% and increased with age from 5.2% in 1–3 year olds, to 9.3% in 7–9 year olds (p = 0.015). In 16 of the 30 hamlets, no children ages 1–3 years had antibodies to pgp3.

**Conclusions:**

The antibody status of the 1–3 year olds indicates low cumulative exposure to infection during the surveillance period. Four years post MDA, there is no evidence for re-emergence of follicular trachoma.

## Author Summary

Trachoma, the leading infectious cause of blindness world-wide, is targeted for elimination by 2020. The World Health Organization advises districts to undertake surveillance for trachoma when follicular trachoma (TF) is less than 5% in children 1–9 years. In a trachoma-endemic district that stopped its program four years ago, we undertook a surveillance survey, adding to the assessment of TF a test for *C*. *trachomatis* infection, and a dried blood spot which was processed for antibodies to *C*. *trachomatis* antigen pgp3; antibody status may indicate cumulative past exposure to infection. The prevalence of TF was 0.4%, below the 5% cut-off indicating that trachoma elimination had been achieved with no re-emergence. The antibody positivity overall was low, 7.5%, and increased with age from 5.2% in 1–3 year olds, to 9.3% in 7–9 year olds (p = 0.015). In 16 of the 30 hamlets, no children ages 1–3 years had antibodies to pgp3. The antibody status of the 1–3 year olds indicated low cumulative exposure to infection during the surveillance period. In summary, four years post -program, there is no evidence for re-emergence of trachoma using any indicator sufficient to cause re-emergence.

## Introduction

Trachoma, the leading infectious cause of blindness world-wide, is caused by repeated episodes of ocular infection with the bacterium *Chlamydia trachomatis* [1]. Trachoma is the target of a massive global control program, from global mapping to country programs working to eliminate blinding trachoma district by district [2–4]. The World Health Organization (WHO) has established Ultimate Intervention Goals as guidance for countries, and included two metrics: (1) reduction in the prevalence of follicular trachoma (TF) in children ages 1–9 to less than 5% at district level, and (2) reduction in the number of cases of trachomatous trichiasis, the late-stage complication where the eyelashes rub the globe, to less than 1/1,000 total population at district level. A population-based impact survey to check the progress of program activities is the recommended monitoring tool [5].

Once an impact survey has documented that a district has achieved a TF prevalence of <5% in children ages 1–9 years, the program can cease antibiotic interventions while is still encouraged to continue with facial hygiene and environmental change activities. The district now enters into a surveillance phase to monitor for re-emergence of the disease. In September 2014, WHO convened a working group which released surveillance guidelines: a single population-based surveillance (“pre-validation”) survey will be carried out at district level, at least two years after the last round of mass drug administration. The guidelines anticipate that re-emergence, if it is to happen, will be evident by two years although re-emergence to what level of TF (>5% or >10% TF for example) has not been defined. The surveillance survey still relies on clinical assessment of trachoma (TF) to determine if the program has succeeded in sustained reduction of trachoma. There is no other accepted measure to use to guide surveillance. A test of infection with *C*. *trachomatis* is highly susceptible to antibiotic pressure, and studies have shown that where the prevalence of trachoma (TF) is high but infection is near zero, there is a risk of re-emergence of infection [6]. Other studies suggest that when infection is re-introduced into low TF prevalence settings, transmission is not sustainable and infection dies out [7,8]. There is no accepted prevalence level of infection that could guide programs on when to cease antibiotics or to indicate a risk of re-emergence of either infection or trachoma (TF).

Recent work on a test for antibodies to *C*. *trachomatis* antigens suggests serology is a promising tool that indicates cumulative risk of exposure to *C*. *trachomatis*[9,10]. If serology can be used in the youngest age groups to monitor evidence of exposure since the cessation of mass antibiotic provision, it may prove to be a useful tool for confirming interruption of transmission. We report data using the new WHO guidelines on trachoma surveillance, supplemented with a test of infection and a serologic test of antibody positivity, from a district-wide, population-based surveillance survey carried out in Kilosa district, Tanzania, four years after program activities were stopped. The goals were two fold: first, to determine the overall prevalence of TF and infection at district level, and second, to determine the relationship of antibody status to prevalence of TF and Infection by age and by community prevalence of infection and/or TF.

## Methods

### Population

Kilosa district, Tanzania, was formerly trachoma-endemic, with village surveys in 2004 averaging 25% trachoma prevalence. Mass drug administration (MDA) using azithromycin was undertaken sporadically in all hamlets, then in earnest throughout the district from 2007–2010. The national program conducted an impact survey at that time and estimated the rate of follicular trachoma (TF) in children ages 1 to 9 years at 4·17%. For the current surveillance survey, all rural hamlets (n = 522) were eligible. The hamlets ranged in population size from 225 to 876.

### Sample

In 2014, we took a simple random sample of 30 hamlets. The hamlets did not have maps or a list of households from which to randomly sample sentinel children, so we used a random walk method to obtain a sample of 50 children ages 1 to 9 years. We went to the center of the hamlet, as identified by the local hamlet leader. From there, the closest house was designated as the direction to start, and a random number between 0 and 9 was picked from a sealed envelope for that hamlet. The survey team proceeded in the chosen direction, going one by one to the house that corresponded to that number. If there were no children ages 1 to 9 years, the team proceeded in the same direction to the next house, going one by one until 50 children were surveyed per hamlet. In the event the team reached the physical end of the hamlet, they turned to the right and proceeded to the next house, then back in the same direction to the center of the hamlet.

### Survey methods

Clinical signs of trachoma were assessed by a grader trained by a senior grader (SW) using the WHO simplified grading scheme [11]. Because trachoma was rare in this setting, the grader was trained using an extensive set of images, and then completed an assessment using the Global Trachoma mapping project set of images which required agreement of kappa = 0.6. For the first five hamlets, any positive case was also reviewed by the senior grader. Since all were confirmed, the grader was left to assess the remaining hamlets. Active trachoma was classified as either follicular trachoma (TF) or intense inflammatory trachoma (TI). In order to detect *C*. *trachomatis* infection, conjunctival swabs were obtained from the right eye of each child. Strict adherence to protocol was observed to avoid field contamination, and control swabs were taken in the field to monitor possible contamination. These were labeled and analyzed in an identical fashion to true specimens. The swabs were placed in a dry tube, kept cold in the field and in Kilosa and sent to the International STD Laboratory at Johns Hopkins University in Baltimore, MD within 30 days of collection. Blood was collected by finger prick from each child onto filter papers with six circular extensions, each calibrated to collect 10 μl of whole blood (TropBio Pty Ltd, Townsville, Queensland, Australia). These were dried and also shipped to the Johns Hopkins University.

### Laboratory methods

The ocular swabs were analyzed by nucleic acid amplification test (NAAT) for presence of ocular *C*. *trachomatis* rRNA usingAptima Combo2 (AC2) (Hologic, San Diego, CA), following manufacturer’s specifications. The lab personnel were masked to ocular and control swabs. A positive swab is considered infection for purposes of this analysis. The blood spots were analyzed for antibody to chlamydial antigen pgp3 as previously reported [9] using a multiplex bead assay on a Luminex 100 platform. Two different couplings of pgp3 to beads were used, necessitating the generation of unique cutoffs for each coupling. The results are reported as median fluorescence intensity minus background (MFI-BG, where background is the signal from beads with buffer only) and the positivity cut-off for the first set was 606, and for the second set was 869 as determined by receiver operator characteristics (ROC) analyses [9]. ROC analyses were done for both bead sets.

### Data analyses

The prevalences of TF, C. *trachomatis* infection (defined as a positive ocular swab), and antibodies against the chlamydial antigen pgp3 in children 1 to 9 were calculated for each community as the number of children positive divided by number of children examined. The overall district prevalences of the three outcomes are estimated as the mean of the individual community prevalences. In order to derive the confidence intervals for the overall prevalence, because the distribution of prevalence of TF and infection with *C*. *trachomatis* were skewed with most communities having zero prevalence we recalculated the estimate using 1000 bootstrap samples to derive the 2·5% and 97·5% percentiles. The proportion of children positive for antibodies against pgp3 is presented for 3 age categories: 1 to <4 years (born after last MDA), 4 to <7 (born during MDA period), and 7 years or older (born before initiation of MDA). The Mantel-Haenzel Chi-Square statistic was used to test for the presence of a linear trend with increasing age.

### Ethical clearance

The study protocol and forms were reviewed and approved by the Johns Hopkins Institutional Review Board and the National Institute for Medical Research in Tanzania. All guardians gave written, informed consent to participation of the children. Children, ages seven and older provided assent.

## Results

**Fig 1:**
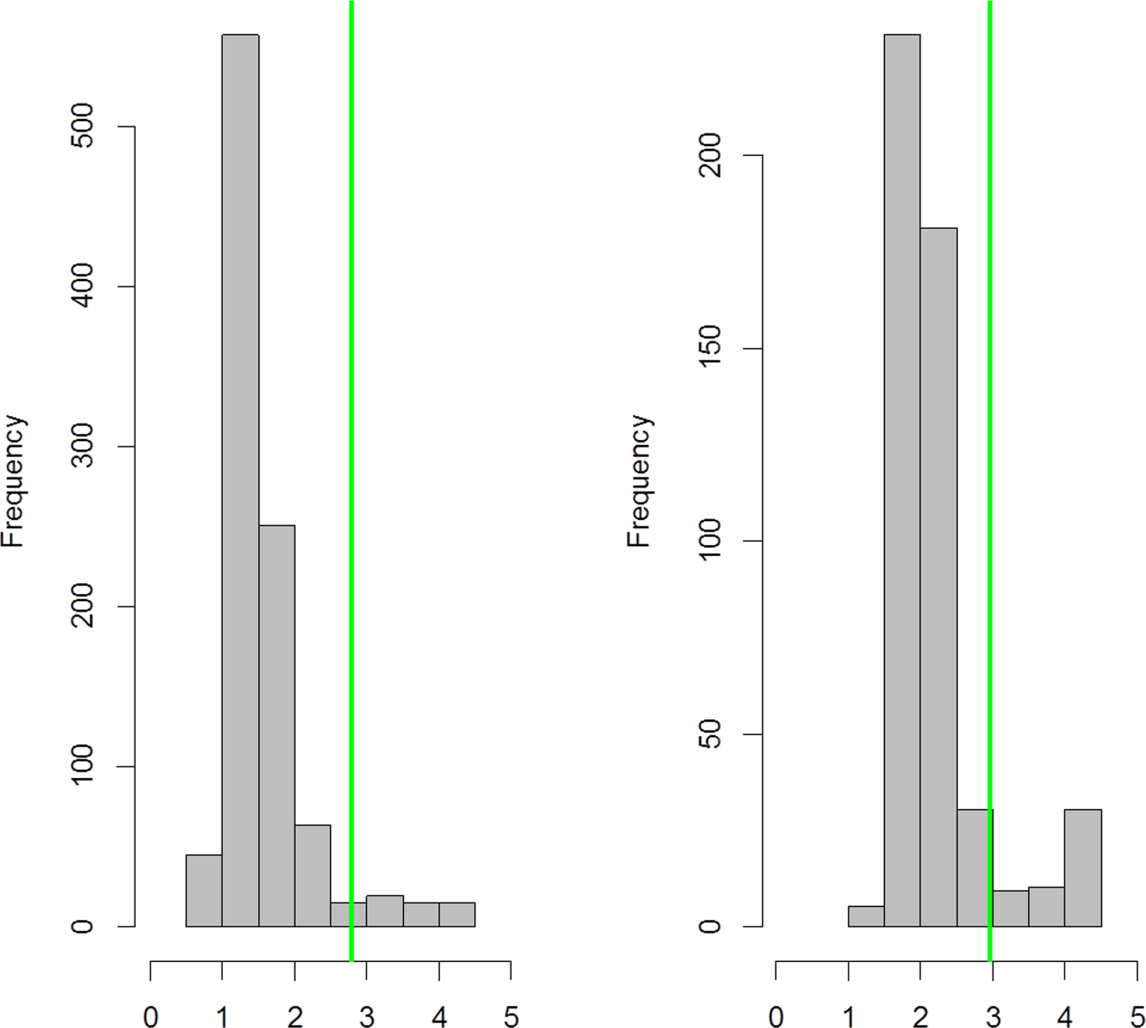
Frequency of antibody levels to the chlamydial antigen pgp3 in 1–9 year olds. Plot shows MFI-BG for antibody responses to pgp3 against the frequency of responders in each grouping of responses from 0 to 10^5^. The x-axis is shown as log _10_ MFI-BG. The green bar shows the cut-offs for each bead set.

A total of 1474 children in 30 communities were enrolled in the study, from the 1501 that were invited (98%). The district prevalence of TF was 0·4% (95% CI = 0·01–0·80), and the prevalence of infection with *C*. *trachomatis* was 1·1% (95% CI = 0.3–2·4). None of the field “air” swabs were positive. The overall prevalence of antibody positivity to pgp3 was 7·5% (95% CI = 5·1–10·1); the overall response to antibody suggests the majority of children had very low levels with only a few having MFI-BG greater than the cut off value (Fig 1). The antibody positivity increased by age group (Table 1). Because we expected that antibody levels in children ages 1–3 years would reflect the period post-program intervention, we further evaluated the characteristics of antibody positivity in that age group in addition to the 1–9 year-old age group.

**Table 1:** The Prevalence of Antibody Positivity by Age group.

Age Group	N	%+pgp3^1^
1–3·9	504	5·2%
4–6·9	528	8·1%
7–9·9	442	9·3%
All Ages	1474	7·5%

^1^ Test for trend p = 0·015

**Fig 2:**
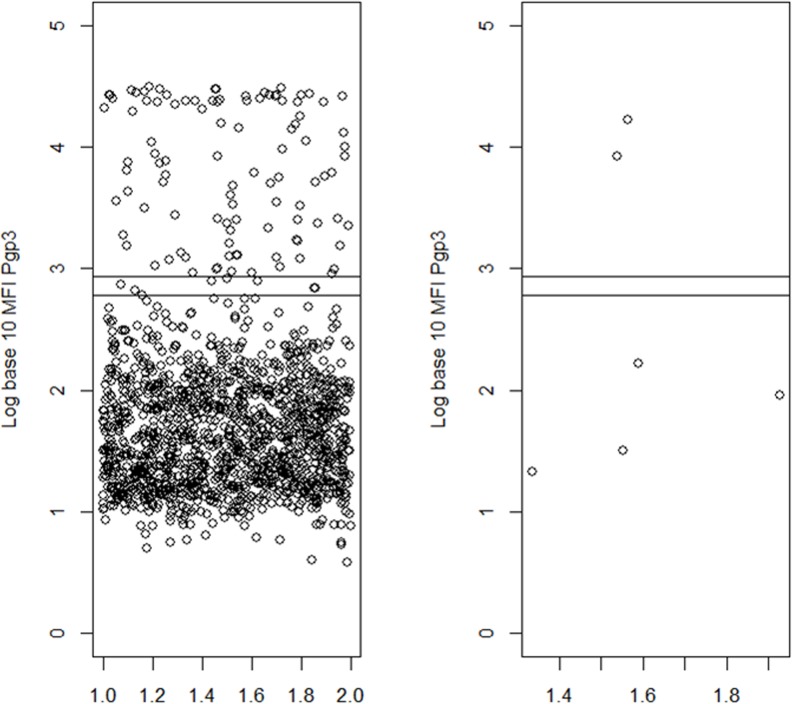
Log of antibody response in children ages 1–9 years with and without Follicular Trachoma (TF). The two bars correspond to the two cut-offs with each bead coupling. The X axis in each group corresponds to the result of a single child.

**Fig 3:**
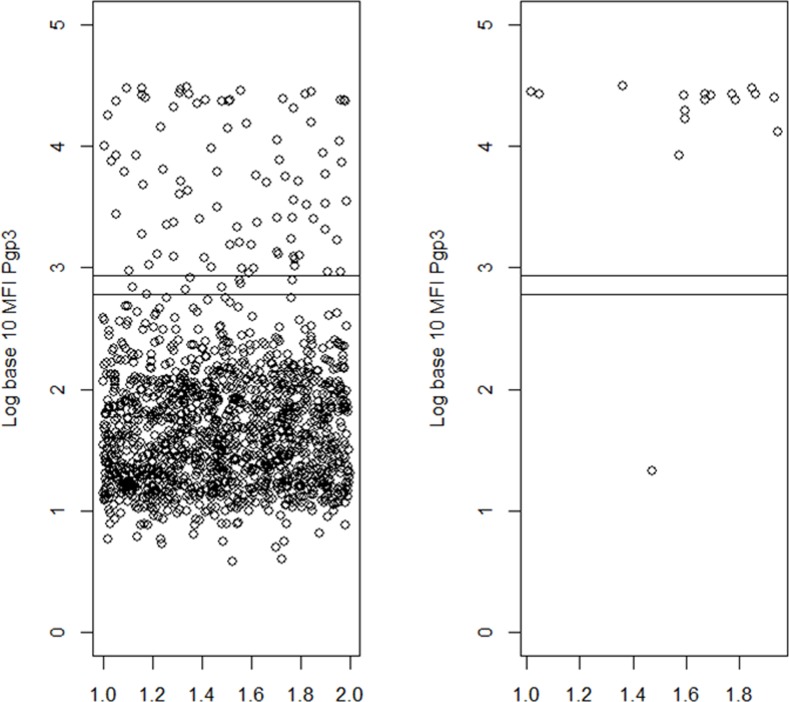
Log of Antibody Response in children ages 1–9 years who have and do not have Infection with C. trachomatis. The two bars correspond to the two cut-offs with each bead coupling. The X axis in each group corresponds to the result of a single child.

The level of antibody positivity according to the TF presence, or presence of infection, in the children is shown in Figs 2 and 3. While most of the TF cases had antibody below the cut offs, all but one of the cases of infection had antibody levels well above cut off.

Eight hamlets had infection or trachoma (TF), or both infection and trachoma (TF), while 22 communities had neither infection nor trachoma (TF). Of the eight hamlets with TF and/or infection, two had both TF and infection, three had infection alone, and three had TF alone (Table 2). TF was low in the five hamlets with TF, between 2% and 3% prevalence (Table 2). The prevalence of Infection was greater than 5% in the few hamlets with infection and TF or infection alone, with 6% and 7% infection rates, respectively, in 1–9 year olds. Overall, antibody positivity among 1–3 year olds—the children born after cessation of MDA—was higher in the hamlets with infection compared to those without (Table 2).

**Table 2:** The prevalence of antibody positivity in children who reside in hamlets with both infection and trachoma (TF), infection alone, trachoma (TF) alone, or neither infection nor trachoma (TF).

Trachoma (TF)/Infection Status of Hamlet	Number of Hamlets	Average %TF in 1–9 year olds	Average % infection in 1–9 year olds	Average %pgp3 in 1–9 year olds	Average %pgp3 in 1–3 year olds
Both infection and TF	2	3·0%	6·0%	11·0%	7·1%
Infection alone	3	0	7·3%	19·2%	10·8%
(TF) alone	3	2·0%	0	6·0%	2·1%
Neither	22	0	0	5·9%	4·4%

**Fig 4:**
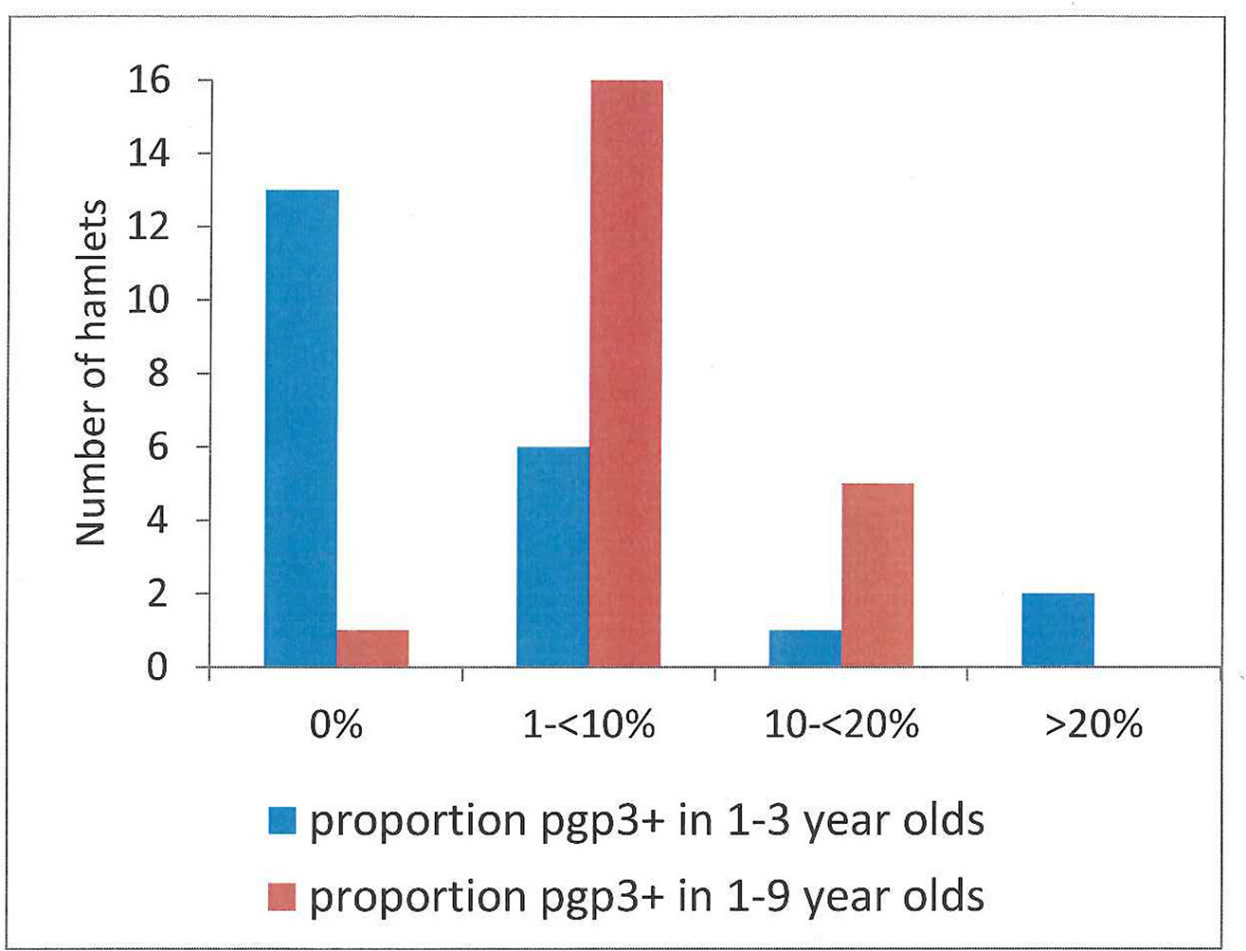
Among the 22 hamlets with neither trachoma nor infection, the number of hamlets with zero proportion positive to pgp3, the number with 1-<10% positive, 10-<20% positive, and 20%+ positive, according to the proportion positive in the age group 1–3 years and in the age group 1–9 years.

A range of antibody-positive rates for children ages 1–3 years was observed within the 22 hamlets that had neither infection nor TF, although 13 of these hamlets (59%) had no individuals with detectable antibody responses. Six hamlets (27%) had seropositivity rates between 1 and<10%, one was between 10 and 20% seropositive, and two (9%) were >20% seropositive for children ages 1–3 years (Fig 4). For children ages 1–9 years, 17 hamlets (77%) had overall antibody-positivity rates <10% (Fig 2).

## Discussion

This is the first “surveillance” survey to be reported for trachoma (TF) using the new World Health Organization interim guidelines. Kilosa district appears to have sustained reduction in TF four years after stopping all trachoma program activities, now at 0·4% TF, consistent with interruption of transmission for most communities. Interestingly, the prevalence of infection, measured using a very sensitive nucleic acid amplification test, was 1·1%, and higher than disease rates, but was concentrated in just a few villages. This was also observed in a previous survey in this district [12]. The antibody-positivity prevalence in two of the villages with the highest rates of infection was greater than 10% in children ages 1–3 years, further supporting ongoing transmission in those villages. However, in three hamlets with neither infection nor TF, the antibody positivity was also above 10%. This was clearly not indicating ongoing transmission of infection, but was likely indicative of past exposure, as we see in other trachoma endemic communities where groups of children who do not have infection or TF have high rates of antibody positivity [10]. We do not expect antibody positivity to be indicative of current infection or transmission, but rather cumulative exposure to *C*. *trachomatis*. In these hamlets, children in the age group 1–3.9 years may have had exposure after the program stopped activity, but do not currently have either infection or disease. Other possible reasons are described below.

A characteristic increase by age in prevalence of antibody positivity against *C*. *trachomatis* antigens was observed, even in this district with virtual absence of trachoma. Previous reports in trachoma-endemic communities have found a similar trend but with a sharper rise in the age prevalence of antibody positivity [9,10]. In this district, the youngest age group, ages 1–3 years, who were born post- MDA and during surveillance, had the lowest prevalence at 5.2%. The 4–7 year olds, born during the last three cycles of yearly rounds of MDA, had the next lowest prevalence, 8·1%. The children who were born before the last MDA cycle began, ages 7–9 years at this survey, had a prevalence of antibody positivity of 9·3%. Pgp3-specific antibody responses were generally quite low in this district. This is in contrast to high seroprevalence seen in previous studies in trachoma endemic villages with ongoing transmission [10]. These responses were low even in comparison to 4 villages in neighboring Kongwa district, where TF prevalence averaged 2.9% and antibody prevalence was 22% in 1–9 year olds (S1 File). Kilosa had already had intermittent mass drug administration over a seven year period prior to 2008 which may have reduced exposure to ocular *C*. *trachomatis* infection even in the oldest children.

The low antibody levels seen in this survey likely reflect the low transmission in Kilosa district, but the heterogeneity across hamlets, and especially the >20% seroprevalence in two hamlets with no infection or trachoma, suggests that other causes of antibodies recognizing pgp3 should also be considered. For example, pgp3-specific antibodies may be generated from ocular or respiratory infection arising from transmission from a mother with genital infection to her newborn during delivery [13,14]. We have no data on genital infection in this district, but it is notable that the two high antibody prevalence villages were close to the main truck road in Kilosa. It is also possible that families that travel for cultural reasons to other, still-endemic, districts in Tanzania may acquire trachoma there and return to Kilosa. A similar finding was observed in The Gambia, where—despite the fact that families returned with infection—transmission was not sustainable and trachoma died out [7]. That phenomenon appears to also be the case in Kilosa where district rates of infection are still low. There are no data on the level of infection required to cause seroconversion, but it may be possible that infection was introduced then died out, but left cases of seroconversion.

There are limitations to the study. Ideally, the determination of antibody status at the time of the impact survey immediately post-MDA would have enabled us to follow the trajectory of antibody responses over the time of surveillance and provided stronger evidence for the assertion that <6% in children ages 1–3 years represents evidence of low transmission. The fact that close to half the sample hamlets had young children with no antibodies to pgp3 is encouraging and suggests that this district has not experienced re-emergence of transmission as determined by antibody testing. Also, the antibody positivity rate is subject to the choice of cut-off for each run. In this study, two different bead couplings necessitated the generation of two separate cut-offs. An international standard, such as a humanized monoclonal antibody against pgp3, would allow direct comparisons between different couplings and analyses done in different settings.

We also recognize that the use of a Luminex platform is expensive, not widely available, and technically complicated. If it is to be used in a program context, further work on development of an ELISA or a field test strip would be more practical. We are working on this aspect, but are encouraged to do so by the data found here at district level.

Finally, the surveillance survey was powered to detect low prevalences at the district level, and there are more uncertainties around the estimates of prevalence within the sub groups. However, the data on prevalences within age groups and within the hamlets organized by prevalences of TF and infection were informative and we felt reasonable to show.

The heterogeneity observed in the different hamlets also points to the importance of interpreting data at the district, not individual village, level. Districts represent a spectrum of their communities’ prevalence of disease, infection, and antibody status. Focusing undue attention on a few seemingly anomalous hamlets with infection would have obscured the general finding that our surveillance survey indicated trachoma (TF) is no longer a public health problem in this district, regardless of the tool we might have used to measure it. Our surveillance survey found, four years after cessation of MDA, that TF had not returned and that a prevalence of less than 6% pgp3 antibody in children ages 1–3 years was associated with absence of re-emergence of trachoma in this district. In fact, these data suggest that any of the assessments used provided a good marker of trachoma elimination.

## Supporting Information

S1 Checklist STROBE Checklist. (DOCX)

S1 File Study of Four Villages in Kongwa. Antibody prevalence by age and Village.
(DOCX)
