# Supplementary material for: Can We Use Antibodies to Chlamydia trachomatis as a Surveillance Tool for National Trachoma Control Programs? Results from a District Survey
Source: PLoS Negl Trop Dis. 2016 Jan 15;10(1):e0004352. doi: 10.1371/journal.pntd.0004352 (PMC4714879; doi:10.1371/journal.pntd.0004352)
Supplement: S1 Checklist — (DOCX) [file pntd.0004352.s001.docx]

STROBE Statement—checklist of items that should be included in reports of observational studies

|  | Item No. | Recommendation | Page  No. | Relevant text from manuscript |
| --- | --- | --- | --- | --- |
| **Title and abstract** | 1 | (*a*) Indicate the study’s design with a commonly used term in the title or the abstract | 1 | Results from a district survey |
|  |  | (*b*) Provide in the abstract an informative and balanced summary of what was done and what was found | 2 | Methodology: We randomly sampled 30 hamlets in Kilosa district, Tanzania, and randomly selected 50 children ages 1–9 per hamlet. The tarsal conjunctivae were graded for trachoma (TF), tested for C. trachomatis infection (Aptima Combo2 assay: Hologic, San Diego, CA), and a dried blood spot processed for antibodies to C. trachomatis pgp3 using a multiplex bead assay on a Luminex 100 platform.  Principal Findings: The prevalence of trachoma (TF) was 0.4%, well below the <5% indicator for re-starting a program. Infection was also low, 1.1%. Of the 30 hamlets, 22 had neither infection nor TF. Antibody positivity overall was low, 7.5% and increased with age from 5.2% in 1–3 year olds, to 9.3% in 7–9 year olds (p=0.015). In 16 of the 30 hamlets, no children ages 1–3 years had antibodies to pgp3. |
| Introduction | | | |  |
| Background/rationale | 2 | Explain the scientific background and rationale for the investigation being reported | 4 | (see entire introduction) |
| Objectives | 3 | State specific objectives, including any prespecified hypotheses | 5 | The goals were two fold: first, to determine the overall prevalence of TF and infection at district level, and second, to determine the relationship of antibody status to prevalence of TF and Infection by age and by community prevalence of infection and/or TF. |
| Methods | | | |  |
| Study design | 4 | Present key elements of study design early in the paper | 6 | Sample: In 2014, we took a simple random sample of 30 hamlets. The hamlets did not have maps or a list of households from which to randomly sample sentinel children, so we used a random walk method to obtain a sample of 50 children ages 1 to 9 years |
| Setting | 5 | Describe the setting, locations, and relevant dates, including periods of recruitment, exposure, follow-up, and data collection | 5-6 | (see methods section) |
| Participants | 6 | (*a*) *Cohort study*—Give the eligibility criteria, and the sources and methods of selection of participants. Describe methods of follow-up  *Case-control study*—Give the eligibility criteria, and the sources and methods of case ascertainment and control selection. Give the rationale for the choice of cases and controls  *Cross-sectional study*—Give the eligibility criteria, and the sources and methods of selection of participants | 6 | we took a simple random sample of 30 hamlets. The hamlets did not have maps or a list of households from which to randomly sample sentinel children, so we used a random walk method to obtain a sample of 50 children ages 1 to 9 years. |
|  |  | (*b*) *Cohort study*—For matched studies, give matching criteria and number of exposed and unexposed  *Case-control study*—For matched studies, give matching criteria and the number of controls per case | n/a |  |
| Variables | 7 | Clearly define all outcomes, exposures, predictors, potential confounders, and effect modifiers. Give diagnostic criteria, if applicable | 6-7 |  |
| Data sources/ measurement | 8* | For each variable of interest, give sources of data and details of methods of assessment (measurement). Describe comparability of assessment methods if there is more than one group | *6-7* |  |
| Bias | 9 | Describe any efforts to address potential sources of bias | 6 | The hamlets did not have maps or a list of households from which to randomly sample sentinel children, so we used a random walk method to obtain a sample of 50 children ages 1 to 9 years. We went to the center of the hamlet, as identified by the local hamlet leader. From there, the closest house was designated as the direction to start, and a random number between 0 and 9 was picked from a sealed envelope for that hamlet. The survey team proceeded in the chosen direction, going one by one to the house that corresponded to that number. If there were no children ages 1 to 9 years, the team proceeded in the same direction to the next house, going one by one until 50 children were surveyed per hamlet. In the event the team reached the physical end of the hamlet, they turned to the right and proceeded to the next house, then back in the same direction to the center of the hamlet. |
| Study size | 10 | Explain how the study size was arrived at | 4 | WHO guidelines for 30 cluster randomized population based survey |

Continued on next page

| Quantitative variables | 11 | Explain how quantitative variables were handled in the analyses. If applicable, describe which groupings were chosen and why | 8 | The prevalences of TF, C. trachomatis infection (defined as a positive ocular swab), and antibodies against the chlamydial antigen pgp3 in children 1 to 9 were calculated for each community as the number of children positive divided by number of children examined. The overall district prevalences of the three outcomes are estimated as the mean of the individual community prevalences. In order to derive the confidence intervals for the overall prevalence, because the distribution of prevalence of TF and infection with C. trachomatis were skewed with most communities having zero prevalence we recalculated the estimate using 1000 bootstrap samples to derive the 2·5% and 97·5% percentiles. The proportion of children positive for antibodies against pgp3 is presented for 3 age categories: 1 to <4 years (born after last MDA), 4 to <7 (born during MDA period), and 7 years or older (born before initiation of MDA). The Mantel-Haenzel Chi-Square statistic was used to test for the presence of a linear trend with increasing age. |
| --- | --- | --- | --- | --- |
| Statistical methods | 12 | (*a*) Describe all statistical methods, including those used to control for confounding | 8 | See above |
|  |  | (*b*) Describe any methods used to examine subgroups and interactions | 8 | See above |
|  |  | (*c*) Explain how missing data were addressed |  | 98% of children participated-there were no missing data |
|  |  | (*d*) *Cohort study*—If applicable, explain how loss to follow-up was addressed  *Case-control study*—If applicable, explain how matching of cases and controls was addressed  *Cross-sectional study*—If applicable, describe analytical methods taking account of sampling strategy | n/a |  |
|  |  | (*e*) Describe any sensitivity analyses |  |  |
| Results | | | | |
| Participants | 13* | (a) Report numbers of individuals at each stage of study—eg numbers potentially eligible, examined for eligibility, confirmed eligible, included in the study, completing follow-up, and analysed | n./a |  |
|  |  | (b) Give reasons for non-participation at each stage | n/a |  |
|  |  | (c) Consider use of a flow diagram | n/a |  |
| Descriptive data | 14* | (a) Give characteristics of study participants (eg demographic, clinical, social) and information on exposures and potential confounders | Table 1, Figures 2 and 3 |  |
|  |  | (b) Indicate number of participants with missing data for each variable of interest | n/a |  |
|  |  | (c) *Cohort study*—Summarise follow-up time (eg, average and total amount) |  |  |
| Outcome data | 15* | *Cohort study*—Report numbers of outcome events or summary measures over time |  |  |
|  |  | *Case-control study—*Report numbers in each exposure category, or summary measures of exposure |  |  |
|  |  | *Cross-sectional study—*Report numbers of outcome events or summary measures | *Table 1* |  |
| Main results | 16 | (*a*) Give unadjusted estimates and, if applicable, confounder-adjusted estimates and their precision (eg, 95% confidence interval). Make clear which confounders were adjusted for and why they were included | 8 | A total of 1474 children in 30 communities were enrolled in the study, from the 1501 that were invited (98%). The district prevalence of TF was 0·4% (95% CI=0·01-0·80), and the prevalence of infection with C. trachomatis was 1·1% (95% CI=0.3-2·4). None of the field “air” swabs were positive. The overall prevalence of antibody positivity to pgp3 was 7·5% (95% CI=5·1-10·1 |
|  |  | (*b*) Report category boundaries when continuous variables were categorized See Table 1 and 2 |  |  |
|  |  | (*c*) If relevant, consider translating estimates of relative risk into absolute risk for a meaningful time period | n/a |  |

Continued on next page

| Other analyses | 17 | Report other analyses done—eg analyses of subgroups and interactions, and sensitivity analyses | As asked by reviewers, graphs 2 and 3 |  |
| --- | --- | --- | --- | --- |
| Discussion | | | | |
| Key results | 18 | Summarise key results with reference to study objectives | 10 | Kilosa district appears to have sustained reduction in TF four years after stopping all trachoma program activities, now at 0·4% TF, consistent with interruption of transmission for most communities. Interestingly, the prevalence of infection, measured using a very sensitive nucleic acid amplification test, was 1·1%, and higher than disease rates, but was concentrated in just a few villages. This was also observed in a previous survey in this district12 |
| Limitations | 19 | Discuss limitations of the study, taking into account sources of potential bias or imprecision. Discuss both direction and magnitude of any potential bias | 12 | Section begins “there are limitations to the study”.. |
| Interpretation | 20 | Give a cautious overall interpretation of results considering objectives, limitations, multiplicity of analyses, results from similar studies, and other relevant evidence | 13 | . Our surveillance survey found, four years after cessation of MDA, that TF had not returned and that a prevalence of less than 6% pgp3 antibody in children ages 1–3 years was associated with elimination of trachoma in this district. In fact, these data suggest that any of the assessments used provided a good marker of trachoma elimination. |
| Generalisability | 21 | Discuss the generalisability (external validity) of the study results | 13 | The heterogeneity observed in the different hamlets also points to the importance of interpreting data at the district, not individual village, level. Districts represent a spectrum of their communities’ prevalence of disease, infection, and antibody status. Focusing undue attention on a few seemingly anomalous hamlets with infection would have obscured the general finding that our surveillance survey indicated trachoma (TF) is no longer a public health problem in this district, regardless of the tool we might have used to measure it. |
| Other information | |  | | |
| Funding | 22 | Give the source of funding and the role of the funders for the present study and, if applicable, for the original study on which the present article is based | Present | We are asked to upload this in a separate file so it is not also in the manuscript |

*Give information separately for cases and controls in case-control studies and, if applicable, for exposed and unexposed groups in cohort and cross-sectional studies.

**Note:** An Explanation and Elaboration article discusses each checklist item and gives methodological background and published examples of transparent reporting. The STROBE checklist is best used in conjunction with this article (freely available on the Web sites of PLoS Medicine at http://www.plosmedicine.org/, Annals of Internal Medicine at http://www.annals.org/, and Epidemiology at http://www.epidem.com/). Information on the STROBE Initiative is available at www.strobe-statement.org.
