# Supplementary material for: Can We Use Antibodies to Chlamydia trachomatis as a Surveillance Tool for National Trachoma Control Programs? Results from a District Survey
Source: PLoS Negl Trop Dis. 2016 Jan 15;10(1):e0004352. doi: 10.1371/journal.pntd.0004352 (PMC4714879; doi:10.1371/journal.pntd.0004352)
Supplement: S1 File — Antibody prevalence by age and Village. (DOCX) [file pntd.0004352.s002.docx]

| Village | Antigen |  | | Prevalence by Age (%) | | | | | | | | |
| --- | --- | --- | --- | --- | --- | --- | --- | --- | --- | --- | --- | --- |
|  |  |  | 1 | | 2 | 3 | 4 | 5 | 6 | 7 | 8 | 9 |
| 0101 | pgp3 |  | 8.3 | | 17.6 | 11.8 | 23.1 | 0 | 28.6 | 37.5 | 50 | 80 |
|  | CT694 |  | 8.3 | | 17.6 | 11.8 | 7.7 | 0 | 28.6 | 37.5 | 50 | 80 |
| 0102 | pgp3 |  | 8.3 | | 9.1 | 10.5 | 42.8 | 25 | 27.3 | 40 | 63.6 | 60 |
|  | CT694 |  | 8.3 | | 18.2 | 10.5 | 35.7 | 12.5 | 36.4 | 40 | 63.6 | 60 |
| 0103 | pgp3 |  | 0 | | 9 | 33 | 18 | 56 | 25 | 75 | 56 | 70 |
|  | CT694 |  | 0 | | 9 | 33 | 9 | 56 | 25 | 75 | 56 | 70 |
| 1303 | pgp3 |  | 0 | | 11 | 15 | 20 | 20 | 33 | 33 | 44 | 50 |
|  | CT694 |  | 0 | | 22 | 0 | 20 | 20 | 33 | 33 | 44 | 43 |
| TOTAL | Both |  | 4.5 (2/44) | | 17.4 (8/46) | 19.3 (12/62) | 32.5 (13/40) | 28.6 (10/35) | 34.2 (13/38) | 45.7 (16/35) | 57.1 (20/35) | 61.5 (24/39) |

Supplemental Information: Study of Four Villages in Kongwa. Antibody prevalence by age and Village.
